# Flattening the curve: Insights from queueing theory

**DOI:** 10.1371/journal.pone.0286501

**Published:** 2023-06-16

**Authors:** Sergio Palomo, Jamol J. Pender, William A. Massey, Robert C. Hampshire

**Affiliations:** 1 Cornell University Systems Engineering, Ithaca, NY, United States of America; 2 Cornell University Operations Research and Information Engineering, Ithaca, NY, United States of America; 3 Princeton University, Operations Research and Financial Engineering, Princeton, NJ, United States of America; 4 University of Michigan, Gerald Ford School of Public Policy, Ann Arbor, MI, United States of America; City University of Hong Kong, HONG KONG

## Abstract

The worldwide outbreak of the coronavirus was first identified in 2019 in Wuhan, China. Since then, the disease has spread worldwide. As it is currently spreading in the United States, policy makers, public health officials and citizens are racing to understand the impact of this virus on the United States healthcare system. They fear a rapid influx of patients overwhelming the healthcare system and leading to unnecessary fatalities. Most countries and states in America have introduced mitigation strategies, such as using social distancing to decrease the rate of newly infected people. This is what is usually meant by *flattening the curve*. In this paper, we use queueing theoretic methods to analyze the time evolution of the number of people hospitalized due to the coronavirus. Given that the rate of new infections varies over time as the pandemic evolves, we model the number of coronavirus patients as a dynamical system based on the theory of infinite server queues with time inhomogeneous Poisson arrival rates. With this model we are able to quantify how flattening the curve affects the peak demand for hospital resources. This allows us to characterize how aggressive societal policy needs to be to avoid overwhelming the capacity of healthcare system. We also demonstrate how curve flattening impacts the elapsed lag between the times of the peak rate of hospitalizations and the peak demand for the hospital resources. Finally, we present empirical evidence from Italy and the United States that supports the insights from our model analysis.

## 1 Introduction

In December of 2019, the coronavirus was first identified in Wuhan. This is a city of over 11 million people in the Hubei province of China. COVID-19, the disease caused by the coronavirus, grew by several thousand per day in China between late January 2020 and early February 2020, the first local peak of their epidemic. The number of daily infections decreased significantly due to stringent containment efforts.

As the viral spread became a global pandemic, large outbreaks in South Korea, Iran, Italy, Spain, and the United States all sparked interest in coronavirus research. There were 219 million cases and 4,510,124 deaths confirmed worldwide, as of September 1^*st*^ 2021. The World Health Organization then officially named COVID-19 a global pandemic. The rapid spread of the virus during that time then led to over 200 countries reporting cases.

Lawmakers and public health experts such as Dr. Anthony Fauci, National Institute of Allergy and Infectious Diseases director, encouraged “social distancing” as a measure to flatten the curve. This implies that we want to reduce the daily peak number of infected people and spread it out over time. Many people understand how flattening the curve impacts the healthcare system from an empirical or qualitative perspective, see [1]. However, there needs to be more *quantitative* research in this area and relatively more research on how much reduction is needed to flatten the curve.

Everyday the hospital has a demand for a finite but reuseable set of shared resources that include beds, ventilators, and medical staff. Our paper models this resource demand as an infinite server queue with a *non-homogeneous Poisson* arrival process and random, mutually independent service times having some common service time distribution. Such a *non-stationary* infinite server model affords us a great deal of modeling flexibility. Examples of what the arrival process can model include

- The daily number of infected people.
- The daily number of hospitalizations.

Moreover, the service times can model, respectively

- The time of being infected.
- The time the patient spends in the hospital.

These infinite server queueing models also describe more realistic complex behaviors such as *multi-modal* patient arrival surges. We can reduce them to the *superposition* of queues with simple uni-modal mean arrival rate functions. We illustrate this type of peak behaviour by using mean arrival rate examples involving *scaled Gaussian* and the *scaled gamma* density functions. Here, we obtain explicit expressions for the peak mean demand rate of the queueing system as well as the time of this peak rate.

Our first goal is to show the global community how the operations research field of non-stationary queueing theory helps us understand the impact of COVID-19 on healthcare systems. Our second goal is to show how leveraging these insights from queues with time-varying rates leads to simple descriptions of COVID-19 pandemic-like hospital patient dynamics. More specifically, we show how to calculate the following metrics:

- The time of the peak infection rate.
- The time of the peak mean number of infected people.
- The *time lag* between them.

We do a similar analysis for the times of locally peak hospitalization rates and the corresponding times with their peak numbers of patients. Both of these results come from studying the same general infinite server queueing model. We also do this analysis explicitly for the cases of scaled Gaussian and gamma density arrival rates. Thus, by using these non-stationary infinite server models, we can demonstrate the impact of public health policies such as social distancing, mask wearing, and curve flattening.

Operationally, what can we learn from situations already observed in China, Europe, and New York City to avoid similar outcomes in other places? From the data, we see that “every hotspot has its own curve”, as shown in Hilk et al [2]. One important advantage of our work is that it can be used to study each individual curve for individual regions around the world. The geographic and temporal clustering of outbreaks have the potential to create a health care system collapse. Many states in the United States have imposed “stay at home” orders to contain the spread of the disease. However, since many people are asymptomatic, we can show through the analysis of our queueing examples that containment measures may take weeks to have an observable effect in the infection data.

Although hospital bed capacity is an important concern for health care officials, it is one of many critical care bottlenecks needing adequate provisioning from limited healthcare resources. After observing many deaths in Italy, many experts and government officials are concerned about the number of available ventilators. Individual ones are needed by patients who become critically ill. Equally important resources include medical specialists and staff members that can supervise the patients in their beds and operate their ventilators properly.

Fundamentally, this paper strives to help healthcare managers understand the optimal number of supplies needed for all these lifesaving resources at the peak of the ultimate curve for their aggregate demand. This paper is for two types of audiences. The first audience, familiar with non-stationary queueing theory, can appreciate the closed form formulas and explicit results for time lags that occur within our models. It is critical that our model is non-stationary since stationary models would not produce such insights. The second audience understands how these models describe the dynamics of infectious disease outbreaks and provide insights about important quantities such as delays and time lags between peak demand or patient arrival rates and peak supply or maximum customer usage. Our goal is to have both audiences benefit from this work.

### 1.1 Main contributions of this paper

We summarize the contributions of this work as follows:

- Derive an expression for the mean number of infected individuals as modelled by an infinite server queue with a non-stationary arrival rate.
- Derive the exact time of the peak load and the value of the peak load for this model.
- Use our peak load calculations to determine the optimal degree of “flattening” needed to make the peak load infection value less than a pre-specified level.
- Study the impact of the duration of the virus on the peak load dynamics of hospital resources and how it affects our flattening policies.

Finally, characterize the nonlinear relationships between

- Flattening the curve.
- The time lag between the peak rate of the newly admitted patients.
- The peak demand for hospital resources.

We also show examples of these time lags being observed in real data.

## 2 Demand for hospital resources: The infinite server queue

In standard epidemiologic models (SEIR), the rate of spread of infection is represented by the reproductive number [3] (2.4 for the alpha strain of COVID), which itself is related to the product of the number of exposures to an infected individual and the probability of infection, if exposed. This leads to an exponential growth model [4]. In a queueing context, this sets the stage for a Poisson arrival distribution. Interventions aimed at reducing the reproductive number focus on either reducing exposures (e.g. lockdowns and closure of public spaces and transportation) or reducing the probability of infection (e.g. social distancing and masking). If the reproductive number is lowered to a value of one, then infections are endemic. Similarly, if this number is below one, then infections decrease. This “flattens” the shape of the curve for the number of infections over time.

Over an infinite time period, the total number of people infected is represented by the area under the curve(s). Examples of things that might change the total number of infections would be reducing the susceptible pool (e.g. vaccination) or reducing the duration of infection, returning people more quickly to the susceptible pool. We do not assume that the “flattened” curve in our model changes the total number infected. By assuming a fixed total number, we are considering how capacity constraints can be addressed solely through changes in the rate of arrival. In this sense, it is a conservative approach which ignores the possibility that capacity relief will occur through any of these other possibilities.

We model the number of infectious patients as an *M*(*t*)/*G*/∞ queueing model. The *M*(*t*) represents a time-varying arrival process, *G* represents a general service distribution and ∞ represents the number of servers. There are two important reasons to begin with this queueing model. First, it is tractable since the distribution is known in closed form for any non-stationary arrival rate. Second, it models the idealized goal of immediate service where no one waits. From the perspective of the COVID-19 epidemic, this means that anyone who wants a bed or a ventilator gets them immediately. These infinite server queues model the maximum *resource demand* for more realistic systems that may have a finite number of servers with impatient customers. Hence, they represent the dynamics of an unconstrained manager having access to an infinite amount of resources. Finally, they have a history of being used to staff finite server systems, (see for example [5–8]) and serve as the first step towards understanding more complex healthcare systems.

### 2.1 The *M*(*t*)/*G*/∞ queue

This section states the closed form formulas for the *M*(*t*)/*G*/∞ queueing model, using the results of [9, 10]. The paper of [9] uses the properties of the Poisson arrival process and Poisson random measures to show that the number in the queue *Q*(*t*), has a Poisson distribution with time varying mean *q*(*t*). As observed in [9, 10], *q*(*t*) has the following explicit solutions

$$\begin{array}{c} q\left(t\right)=E\left[Q\left(t\right)\right]=E[\int_{t-S}^{t}\lambda \left(u\right)\;du]=E\left[\lambda \left(t-S_{e}\right)\right]\cdot E\left[S\right], \end{array}$$

where λ(*u*) is the time varying arrival rate at time *u* ≤ *t*. The random variables *S* and *S*_e_ represent, respectively, the customer service time and its long term residual service time. The distribution for *S*_e_ is called the *stationary excess distribution* and is defined to be

$$\begin{array}{c} P\left\{S_{e}\leq t\right\}\equiv \frac{1}{E[S]}\int_{0}^{t}P\left\{S>u\right\}du,\;\;\;\text{for}\;\text{all}\;t\geq 0. \end{array}$$

Observe that assuming the distribution for *S* is exponential is equivalent to stating that *S* and *S*_e_ have the same distribution. Moreover, saying that *S* is a constant is equivalent to *S*_*e*_ being uniformly distributed over a fixed finite positive interval starting at zero. Also note that [11] shows that the first moment of *S*_e_ can be expressed in terms of the first and second moments of *S* since

$$\begin{array}{c} E\left[S_{e}\right]=\frac{E[S^{2}]}{2E[S]}=\frac{\text{Var}[S]}{2E[S]}+\frac{E[S]}{2}\geq \frac{E[S]}{2}. \end{array}$$

This lower bound for E[*S*_e_] is tight since it can be attained whenever *S* is a constant.

A special case for time-varying arrival rates is when λ(*t*) = 0 for all *t* ≤ 0 and *Q*(0) = 0. The latter condition is a degenerate case for a Poisson distribution and we obtain the formulas

$$\begin{array}{c} q\left(t\right)=E\left[Q\left(t\right)\right]=E[\int_{{\left(t-S\right)}^{+}}^{t}\lambda \left(u\right)\;du]=E\left[\lambda \left(t-S_{e}\right);S_{e}\leq t\right]\cdot E\left[S\right]. \end{array}$$

Hence we only need to make use of this arrival rate function when it is defined on the positive real line.

Finally, as a point of emphasis, note that the word “curve” has two distinct usages:

- Arrival rate functions or service demand.
- Mean queue lengths, queueing loads, or resource demand.

When using infinite server queues to model hospital operations, customer demand maps over to the daily infection rate and resource demand maps over to the daily number of hospital patients. Moreover, there is a causal relationship between the two curves, i.e. you need to flatten the customer demand in order to flatten the resource demand.

Also note that our definition of flattening the curve differs from its typical media usage. In our setting, the average number of people infected over the duration of the epidemic equals a fixed finite number. The resulting peak infection rate is lower in the flattened case and more spread out. We want to understand the capacity usage under different arrival rate shapes. This is a worst-case analysis since a more realistic scenario would have curve flattening leading to an overall lowering of infection numbers.

### 2.2 The *M*_*φ*_(*t*)/*G*/∞ queue with a scaled Gaussian density arrival rate

In this section, we describe the dynamics of the *M*_*φ*_(*t*)/*G*/∞ queue whose arrival rate is driven by the Gaussian density function as shown below:

$$\begin{array}{c} \lambda_{\varphi }\left(t\right)\equiv \frac{\lambda_{\varphi }^{*}}{\sigma }\cdot \varphi (\frac{t-\tau }{\sigma })\;\;\text{where}\;\;\varphi (x)\equiv \frac{1}{\sqrt{2\pi }}\cdot e^{-x^{2}/2}\;\;\text{and}\;\;\underset{-\infty <x<\infty }{\text{max}}\varphi (x)=\frac{1}{\sqrt{2\pi }}. \end{array}$$ (1)

Given the properties of a Gaussian distribution, *τ* is the location in time for the peak arrival rate, and *σ* is the standard deviation from this peak time. Thus, $\lambda_{\phi }^{*}$ equals the total mean number of infected individuals over the lifetime of the epidemic and the peak arrival rate is

$$\begin{array}{c} \underset{-\infty <t<\infty }{\text{max}}\lambda_{\varphi }(t)=\frac{\lambda_{\varphi }^{*}}{\sigma \sqrt{2\pi }}. \end{array}$$

The modelling interpretations of $\lambda_{\phi }^{*}$, *τ*, and *σ* are summarized by the following integral relationships:

$$\begin{array}{c} \lambda_{\varphi }^{*}=\int_{-\infty }^{\infty }\lambda_{\varphi }\left(t\right)dt,\;\;\tau =\frac{1}{\lambda_{\varphi }^{*}}\int_{-\infty }^{\infty }t\cdot \lambda_{\varphi }\left(t\right)\;dt,\;\;\text{and}\;\;\sigma^{2}=\frac{1}{\lambda_{\varphi }^{*}}\int_{-\infty }^{\infty }{\left(t-\tau \right)}^{2}\cdot \lambda_{\varphi }\left(t\right)\;dt. \end{array}$$

**Fig 1:**
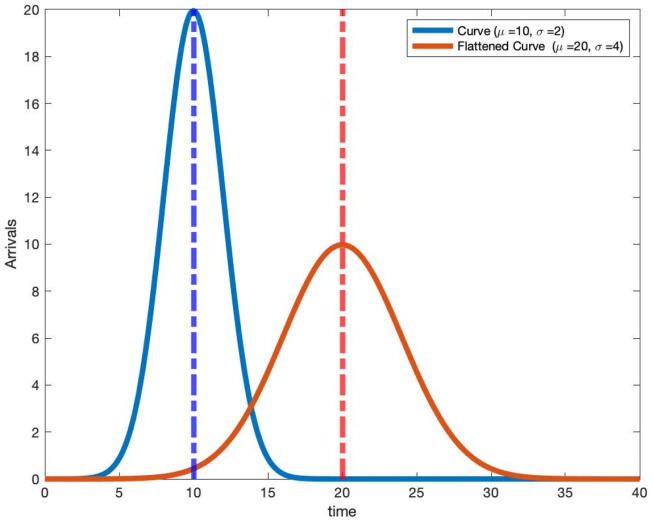
Plot of scaled Gaussian density arrival rate functions.

In Fig 1, we plot two scaled Gaussian density arrival rate functions. The blue curve is a scaled Gaussian(*τ* = 10, *σ* = 2) density arrival function and the red curve (flattened curve) corresponds to the other scaled Gaussian(*τ* = 20, *σ* = 4) density. Increasing the standard deviation by a factor of 2 reduces the peak value of the arrival rate by the same factor. This follows from the peak value of Gaussian being inversely proportional to the standard deviation *σ*. Our increased understanding of this scaled Gaussian density arrival rate function and using it to model hospital patient arrivals, coupled with insights from these non-stationary queues, leads to new insights into the dynamics for the total number of people infected and how long they stay hospitalized.

**Theorem 2.1**. *The queue length of an M*_*φ*_(*t*)/*G*/∞ *queue has a Poisson distribution with mean q*(*t*), *which is the solution to the following ordinary differential equation*

$$\begin{array}{cc} \overset{•}{q}\left(t\right)= & -\frac{\lambda_{\varphi }^{*}}{\sigma^{2}}\cdot E[\frac{t-\tau -S_{e}}{\sigma }\cdot \varphi (\frac{t-\tau -S_{e}}{\sigma })]\cdot E\left[S\right]\\ = & -\frac{q(t)}{\sigma }\cdot E[\frac{t-\tau -S_{e}}{\sigma }\cdot \varphi (\frac{t-\tau -S_{e}}{\sigma })]\cdot E{\left[\varphi (\frac{t-\tau -S_{e}}{\sigma })\right]}^{-1}. \end{array}$$ (2)

*The solution is given by*

$$\begin{array}{c} q\left(t\right)=\frac{\lambda_{\varphi }^{*}}{\sigma }\cdot E[\varphi (\frac{t-\tau -S_{e}}{\sigma })]\cdot E\left[S\right] \end{array}$$ (3)

*and for any value of t*, *we have that*

$$\begin{array}{c} q\left(t\right)\leq \underset{-\infty <t<\infty }{\text{max}}q\left(t\right)=\frac{\lambda_{\varphi }^{*}\cdot E\left[S\right]}{\sigma \sqrt{2\pi }}. \end{array}$$

*Proof*. We actually start with the solution of the queue length given in Eq 3. The solution is easily given by the formula from [9] since we have that

$$\begin{array}{c} q\left(t\right)=E[\lambda_{\varphi }\left(t-S_{e}\right)]\cdot E\left[S\right]=\frac{\lambda_{\varphi }^{*}}{\sigma }\cdot E[\varphi (\frac{t-\tau -S_{e}}{\sigma })]\cdot E\left[S\right]. \end{array}$$ (4)

To find the differential equation, take the derivative of the solution with respect to the time parameter *t*. The final expression in Eq 4 follows from the derivative of the standard Gaussian distribution yielding the first Hermite polynomial times itself i.e. *φ*′(*x*) = −*x*⋅*φ*(*x*). Finally, the tight upper bound on the standard Gaussian density function yields similar bounds for the mean queue length. This completes the proof.

**Fig 2:**
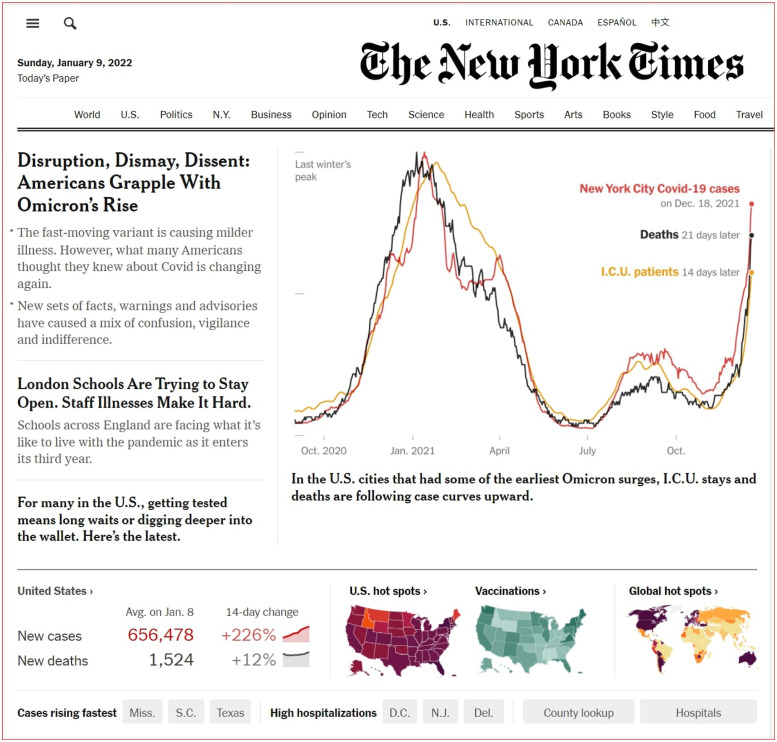
The time lag effect via the New York times data.

**Fig 3:**
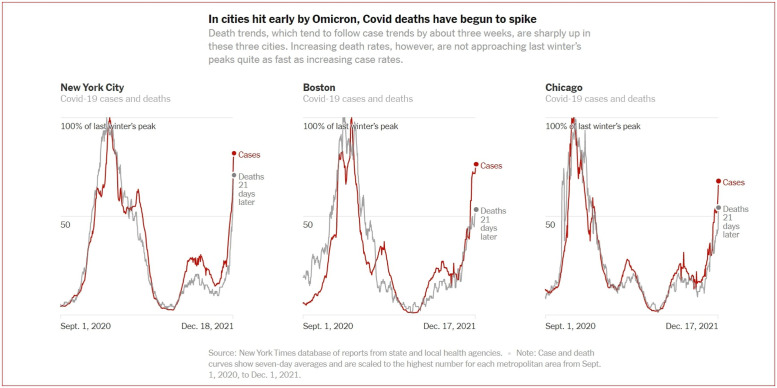
The time lag effect via the New York times data.

We can compute an expression for the time of occurrence of the peak mean queue length. In this paper, we use the time of the peak mean queue length of our model to represent this time. We know that this time for the peak arrival rate occurs at the mode *τ*. Our goal is to understand the *time lag effect*. We define this from when the peak arrival rate occurs until the time of the peak mean queue length. Theorem 2.2 suggests that the time of the peak mean queue length occurs after the peak arrival time *τ*. This well known principle in the queueing literature becomes an important predictor within the dynamics for the COVID-19-like outbreak. These results help to determine exactly from the peak number of infections, when the peak load occurs in healthcare systems. The New York Times article [12] has observed in Fig 2 an empirical time lag effect within COVID-19 data. Moreover, this time lag effect persists throughout the empirical data analysis for citywide infection and ICU admissions rates as well. This is seen in Fig 3.

**Theorem 2.2**. *The time of the peak mean queue length is the solution to the following fixed point equation*

$$\begin{array}{c} t^{*}=\tau +E[S_{e}\cdot \varphi (\frac{t^{*}-\tau -S_{e}}{\sigma })]\cdot E{\left[\varphi (\frac{t^{*}-\tau -S_{e}}{\sigma })\right]}^{-1}, \end{array}$$

*which is equivalent to the equation*

$$\begin{array}{c} t^{*}=\tau +\frac{\lambda_{\varphi }^{*}\cdot E\left[S\right]}{\sigma \cdot q(t^{*})}\cdot E[S_{e}\cdot \varphi (\frac{t^{*}-\tau -S_{e}}{\sigma })]. \end{array}$$

*Proof*. The proofs are found in the Appendix.

**Theorem 2.3**. *The time of occurrence of the peak mean queue length is bounded by the following upper and lower bounds*

$$\begin{array}{c} \tau \leq t^{*}\leq \tau +\frac{E[S_{e}]}{\sigma \sqrt{2\pi }}\cdot E{\left[\varphi (\frac{t^{*}-\tau -S_{e}}{\sigma })\right]}^{-1}. \end{array}$$

*Proof*. Using the first fixed point equation from Theorem 2.2, observe that the numerator is bounded by the standard Gaussian bound given in Eq 1.

Theorem 2.3 provides upper and lower bounds on the time of the occurrence of the peak mean queue length. We can then use a bisection algorithm to determine the time of peak infections exactly. Once the time of the peak infection rate is known, Theorem 2.1 determines both the mean queue length value at that peak time and the value of the peak infection rate. These results provide estimates on the number of ventilators or beds needed at these times.

Now let *C* represent this maximum value for the mean queue length at the time of peak infections. With this maximum mean queue length bound, we now show how to flatten the maximum mean queue length of an *M*_*φ*_(*t*)/*G*/∞ queue using the parameter *σ*. This is the appropriate parameter to use to control the variability with the total mean number of infected staying constant.

**Theorem 2.4**. *Let C denote the maximum of the mean queue length of an M*_*φ*_(*t*)/*G*/∞. *The choice of parameter σ that achieves this value for C is given by the formula*:

$$\begin{array}{c} \sigma^{*}\left(C\right)\equiv \frac{\lambda_{\varphi }^{*}\cdot E\left[S\right]}{C\sqrt{2\pi }}. \end{array}$$

*Proof*. It follows from the standard Gaussian density function that

$$\begin{array}{c} \underset{-\infty <t<\infty }{\text{max}}q\left(t\right)\leq \frac{\lambda_{\varphi }^{*}\cdot E\left[S\right]}{\sigma \sqrt{2\pi }}. \end{array}$$

Setting this upper bound for the mean queue length equal to the value *C* and solving for the standard deviation gives us σ*(*C*).

Since properly initialized *M*(*t*)/*G*/∞ queues always have a Poisson distribution, the following probability is approximately

$$\begin{array}{c} P\{Q\left(t^{*}\right)>E\left[Q\left(t^{*}\right)\right]+{\bar{\Phi }}^{\;-1}\left(\varepsilon \right)\cdot \sqrt{E[Q\left(t^{*}\right)]}\}=P\{\frac{Q\left(t^{*}\right)-E\left[Q\left(t^{*}\right)\right]}{\sqrt{E[Q\left(t^{*}\right)]}}>{\bar{\Phi }}^{\;-1}\left(\varepsilon \right)\}\approx \varepsilon , \end{array}$$

courtesy of the central limit theorem. We call this lower bound for *Q*(t*) an approximate *ε*-*threshold capacity*. It is an *upper bound* for *Q*(t*) with an approximate probability of 1 − *ϵ* at least. Moreover *ε* ≤ 1/2 implies that ${\bar{\Phi }}^{\;-1}\left(\varepsilon \right)\geq 0$, hence

$$\begin{array}{cc} P\{Q\left(t^{*}\right)>\frac{\lambda_{\varphi }^{*}\cdot E\left[S\right]}{C\sqrt{2\pi }} & +{\bar{\Phi }}^{\;-1}\left(\varepsilon \right)\cdot \sqrt{\frac{\lambda_{\varphi }^{*}\cdot E\left[S\right]}{C\sqrt{2\pi }}}\}\\ & \leq P\{Q\left(t^{*}\right)>E\left[Q\left(t^{*}\right)\right]+{\bar{\Phi }}^{\;-1}\left(\varepsilon \right)\cdot \sqrt{E[Q\left(t^{*}\right)]}\}. \end{array}$$

Combining these results gives us the following theorem.

**Theorem 2.5**. *Given some value C*, *an approximate ε*-*threshold flattening for an M*_*φ*_(*t*)/*G*/∞ *queue can be achieved by setting the parameter σ equal to*

$$\begin{array}{c} \sigma^{*}\left(C,\varepsilon \right)=\frac{\lambda_{\varphi }^{*}\cdot E\left[S\right]}{\sqrt{2\pi }}\cdot {\left(\frac{{\bar{\Phi }}^{\;-1}\left(\varepsilon \right)+\sqrt{{\bar{\Phi }}^{\;-1}{\left(\varepsilon \right)}^{2}+4C}}{2C}\right)}^{2}. \end{array}$$

*Proof*. Define *x* to equal the following constant

$$\begin{array}{c} x\equiv \sqrt{\frac{\sigma^{*}\left(C,\varepsilon \right)\cdot \sqrt{2\pi }}{\lambda_{\varphi }^{*}\cdot E\left[S\right]}}=\frac{{\bar{\Phi }}^{\;-1}\left(\varepsilon \right)+\sqrt{{\bar{\Phi }}^{\;-1}{\left(\varepsilon \right)}^{2}+4C}}{2C}. \end{array}$$

Using the ratio of the last term, we can infer from the quadratic formula that *x* is a strictly positive real root for the polynomial equation

$$\begin{array}{c} C\cdot x^{2}-{\bar{\Phi }}^{\;-1}\left(\varepsilon \right)\cdot x-1=0\;\;\;\text{or}\;\text{equivalently}\;\;\;C=\frac{1}{x^{2}}+{\bar{\Phi }}^{\;-1}\left(\varepsilon \right)\cdot \frac{1}{x}. \end{array}$$

The latter equation is the *ε*-threshold formula

$$\begin{array}{c} C=\frac{\lambda_{\varphi }^{*}\cdot E\left[S\right]}{\sigma^{*}\left(C,\varepsilon \right)\cdot \sqrt{2\pi }}+{\bar{\Phi }}^{\;-1}\left(\varepsilon \right)\cdot \sqrt{\frac{\lambda_{\varphi }^{*}\cdot E\left[S\right]}{\sigma^{*}\left(C,\varepsilon \right)\cdot \sqrt{2\pi }}} \end{array}$$

and completes the proof.

Note that when *ε* = 1/2, we have ${\bar{\Phi }}^{\;-1}\left(1/2\right)=0$. We can then recover the mean result given in Theorem 2.4. Moreover, as *ε* gets smaller, we need more flattening to achieve the desired capacity results. Finally, note that this formula also provides an approximate prediction interval for the actual stochastic queue length based on controlling the parameter *σ*.

#### 2.2.1 Deterministic service distribution

In this section, we specify the service distribution to be a constant. This assumption simplifies many of the our equations to yield simpler insights. Our first result shows the mean queue length can be described by an ordinary differential equation (ode) and the solution is simply an integral of the arrival rate from the current time minus the constant service rate until the current time. Finally, we also show that the time lag between the peak arrival rate and peak load is equal to half of the service time.

**Corollary 2.6** *The mean queue length of an M*_*φ*_(*t*)/*D*/∞ *is the solution to the ordinary differential equation*

$$\begin{array}{c} \overset{•}{q}\left(t\right)=\lambda_{\varphi }\left(t\right)-\lambda_{\varphi }\left(t-\Delta \right)=\frac{\lambda_{\varphi }^{*}}{\sigma }\cdot \varphi (\frac{t-\tau }{\sigma })-\frac{\lambda_{\varphi }^{*}}{\sigma }\cdot \varphi (\frac{t-\tau -\Delta }{\sigma }) \end{array}$$ (5)

*and the solution is given by*

$$\begin{array}{c} q\left(t\right)=\int_{t-\Delta }^{t}\frac{\lambda_{\varphi }^{*}}{\sigma }\cdot \varphi (\frac{s-\tau }{\sigma })ds=\lambda_{\varphi }^{*}\cdot (\Phi (\frac{t-\tau }{\sigma })-\Phi (\frac{t-\tau -\Delta }{\sigma })). \end{array}$$ (6)

*Finally, the time of the peak is equal to*

$$\begin{array}{c} t^{*}=\tau +\frac{\Delta }{2}. \end{array}$$

*Proof*. This follows from standard analysis and the symmetry of the Gaussian distribution. It is also important to note that the time derivative of the mean queue length is equal to zero at the midpoint *Δ*/2.

**Corollary 2.7**. *The mean queue length for a mixture of M*_*φ*_(*t*)/*D*/∞ *queueing models has the following formula*

$$\begin{array}{c} q\left(t\right)=\sum_{j=1}^{N}p_{j}\lambda_{j}^{*}\cdot (\Phi (\frac{t-\tau_{j}}{\sigma_{j}})-\Phi (\frac{t-\tau_{j}-\Delta }{\sigma_{j}})) \end{array}$$ (7)

*where the* $p_{j}^{\prime }s$ *are the weights of each mixture distribution*.

*Proof*. This follows immediately from the thinning property for non-homogeneous Poisson process.

#### 2.2.2 Exponential service distribution

When the service time distribution is exponential, the mean queue length *q*(*t*) at time *t*, solves the autonomous ordinary differential equation

$$\begin{array}{c} -0.4pt\overset{•}{q}\left(t\right)=\lambda_{\varphi }\left(t\right)-\mu \cdot q\left(t\right). \end{array}$$

This linear ordinary differential equation has a closed form solution given by

$$\begin{array}{c} q\left(t\right)=q\left(0\right)\cdot e^{-\mu t}+\int_{0}^{t}\lambda_{\varphi }\left(s\right)\cdot e^{-\mu \cdot (t-s)}ds. \end{array}$$

**Corollary 2.8** *The M*_*φ*_(*t*)/*M*/∞ *queue has the following closed form expression for its transient mean queue length*

$$\begin{array}{ccc} q(t) & = & \lambda_{\varphi }^{*}\cdot e^{-\mu t}\cdot e^{\mu^{2}\sigma^{2}/2}\cdot \Phi (\frac{t}{\sigma }-\mu \sigma ). \end{array}$$

*Proof*.

$$\begin{array}{ccc} q(t) & = & E[\lambda_{\varphi }\left(t-\tau -S_{e}\right)]\cdot E\left[S\right]\\ & = & \frac{\lambda_{\varphi }^{*}}{\sigma }\cdot \mu \;\sigma \cdot e^{-\mu (t-\tau )}\cdot e^{\mu^{2}\sigma^{2}/2}\cdot \Phi (\frac{t-\tau }{\sigma }-\mu \;\sigma )\cdot \frac{1}{\mu }\\ & = & \lambda_{\varphi }^{*}\cdot e^{-\mu \cdot (t-\tau )}\cdot e^{\mu^{2}\sigma^{2}/2}\cdot \Phi (\frac{t-\tau }{\sigma }-\mu \;\sigma ). \end{array}$$

**Corollary 2.9**. *The mean queue length for a mixture of M*_*φ*_(*t*)/*M*/∞ *queueing models has the following formula*

$$\begin{array}{ccc} q(t) & = & \sum_{j=1}^{N}p_{j}\lambda_{j}^{*}e^{-\mu_{j}\left(t-\tau_{j}\right)}\cdot e^{\mu_{j}^{2}\sigma_{j}^{2}/2}\cdot \Phi (\frac{t-\tau_{j}}{\sigma_{j}}-\mu_{j}\sigma_{j}) \end{array}$$ (8)

*where the* $p_{j}^{\prime }s$ *are the weights of each mixture distribution*.

**Theorem 2.10**. *When the arrival rate function is a scaled Gaussian density and the service time has an exponential distribution, then the lag *ℓ* for the time between the peak arrival rate and the location of the peak mean queue length is given by*

$$\begin{array}{c} \ell \equiv t^{*}-\tau =\sigma \cdot \left(\mu \sigma +\psi \left(\mu \sigma \right)\right), \end{array}$$

*where*

$$\begin{array}{c} \psi^{-1}\left(x\right)\equiv \frac{\varphi (x)}{\Phi (x)}. \end{array}$$

*Proof*. The proof is given in the Appendix in Section 6.1.3.

Thus, for the exponential distribution, we have an explicit formula for the time lag in terms of the *ψ*(⋅) function. This function is related to conditional expectations of Gaussian random variables and is related to the inverse Mills ratio i.e

$$\begin{array}{c} \psi^{-1}\left(x\right)=\frac{\varphi (x)}{\Phi (x)}=E[\;G\;|\;x+G\geq 0\;]=\frac{E[\;G\;;\;x+G\geq 0\;]}{P\{\;x+G\geq 0\;\}}\equiv \frac{E[\;G\;\cdot \{\;x+G\geq 0\;\}]}{P\{\;x+G\geq 0\;\}}, \end{array}$$

where G is a standard (mean zero, variance one) Gaussian random variable. In the context of operations research, the *ψ*(⋅) function has been analyzed in resource sharing settings in [13, 14], as well as, in the context of using risk measures for staffing multi-server queueing systems in [15].

**Fig 4:**
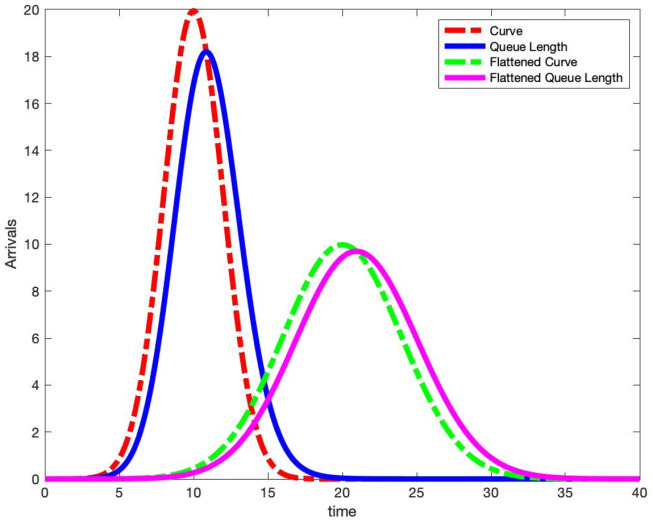
Scaled Gaussian density arrival curves and their mean queue lengths ($E\left[S\right]=1,\lambda_{\phi }^{*}=100$ and exponential service distributions).

In Fig 4, we plot the same two scaled Gaussian density arrival rate functions given in Fig 1. However, to the right of each arrival curve, we also plot the subsequent mean queue length assuming an exponential service distribution. The parameters for each curve and the service distributions are given in Table 1. Table 1 and Fig 4 yield many observations.

**Table 1:** Comparison between regular and flattened curves peak values and times (λ* = 100) and exponential service distributions.

Curve Type	*τ*	*σ*	E[*S*]	Peak Time	Peak Value
Arrival Curve	10	2	1	10	19.95
Flattened Arrival Curve	20	4	1	20	9.97
Mean Queue Length	10	2	1	10.86	18.20
Flattened Mean Queue Length	20	4	1	20.95	9.70

First, note that both arrival rate curves, scaled by the mean service time 1/*μ*, intersect with their mean queue length curves at the time for their peak mean queue length. Since we know the time derivative of the mean queue length is equal to zero this time, then

$$\begin{array}{c} \overset{•}{q}\left(t\right)=\lambda_{\varphi }\left(t\right)-\mu \cdot q\left(t\right)=0 \end{array}$$

implies at the time t* of the occurrence of the peak mean queue length, we must have

$$\begin{array}{c} \lambda_{\varphi }\left(t^{*}\right)=\mu \cdot q\left(t^{*}\right). \end{array}$$ (9)

Second, for the case of E[*S*] = 1, the peak arrival rate is larger than the peak mean queue length. For the flattened arrival curve, its peak mean queue length value is closer to the peak arrival rate than in the non-flattened case. This implies that flattening the curve and reducing the peak arrival rate by one half, does not necessarily reduce the peak mean queue length by one half. For this example, the peak mean queue length is 91% of the peak arrival rate in the non-flattened curve, while it is 97% of the flattened curve.

**Fig 5:**
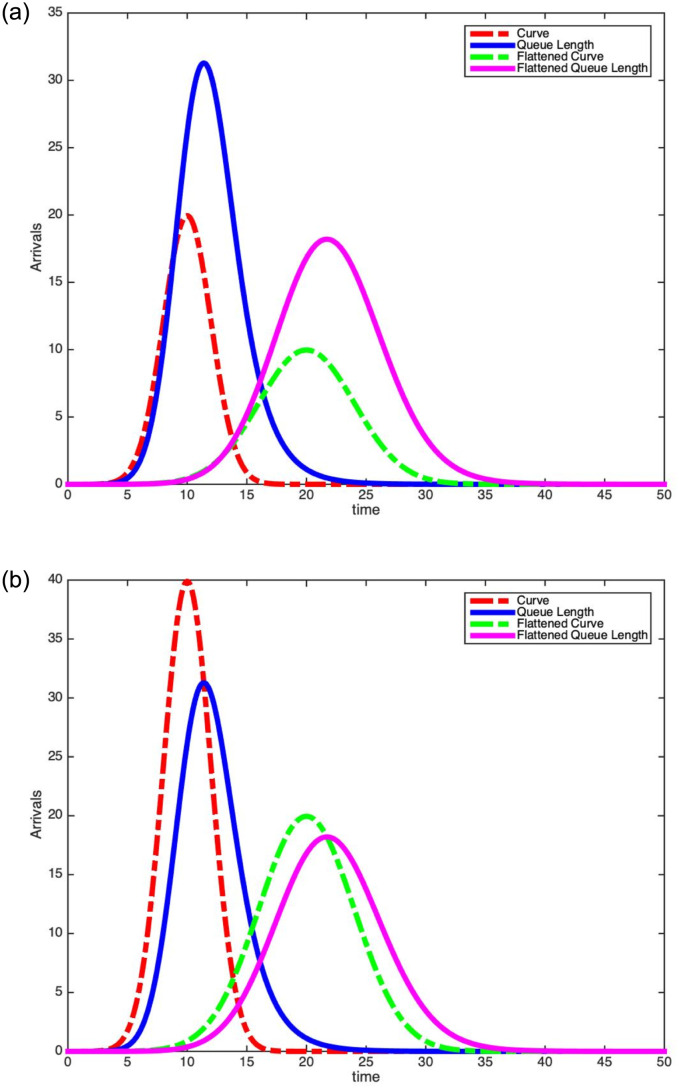
Scaled Gaussian density arrival curves and their mean queue lengths ($E\left[S\right]=2,\lambda_{\phi }^{*}=100$). Arrival Rate (Left). Rescaled Arrival Rate (Right).

**Fig 6:**
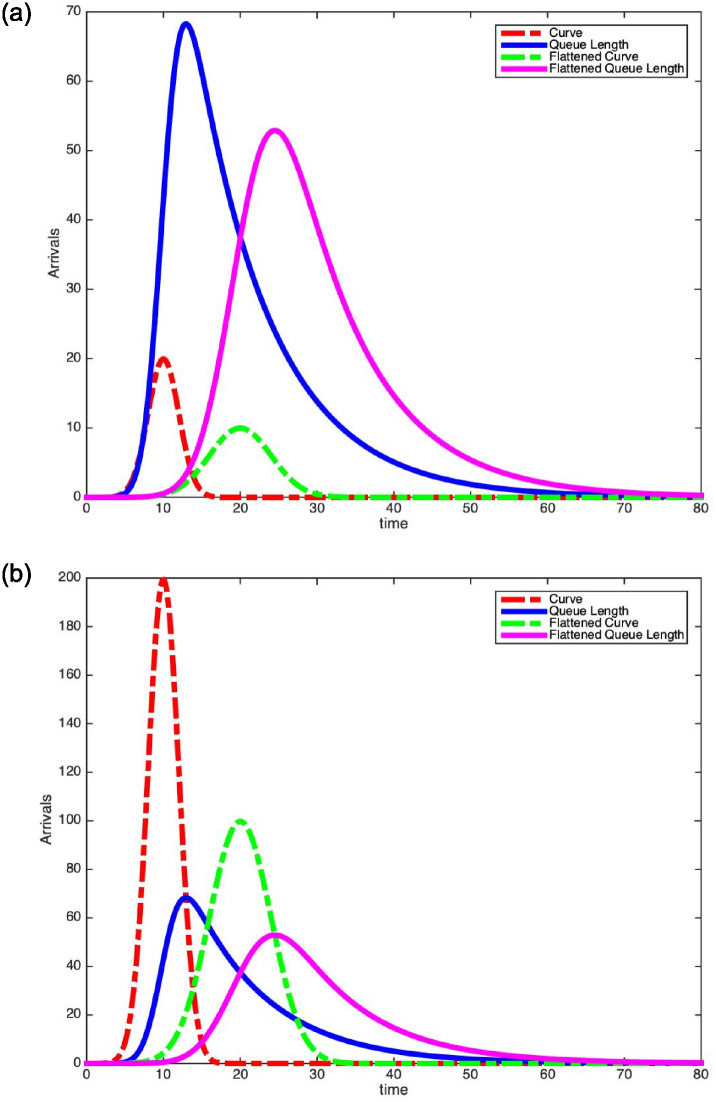
$\lambda_{\phi }^{*}=100,\mu =.1,\sigma =2,\sigma =4,\tau =10,\tau =20$ . Arrival Rate (Left). Rescaled Arrival Rate (Right).

In Figs 5 and 6, we plot our Scaled Gaussian density arrival curves with their respective mean queue lengths. In Fig 5, we set the mean service time to be E[*S*] = 2 and in Fig 6, we set the mean service time to be E[*S*] = 10. On the left-hand sides of Figs 5 and 6, we plot the actual arrival rate and the actual mean queue length. However, on the right-hand sides of Figs 5 and 6, we plot the actual mean queue length, but with an arrival rate that is *rescaled* by the mean service time E[*S*] i.e. λ(*t*) ⋅ E[*S*]. We observe that for the plots on the left, the arrival rate does not intersect with the mean queue length at the peak. However, for the rescaled plots on the right, observe that the arrival rate intersects with the mean queue length curve at the peak of the latter. This verifies Eq 9 again. Finally, we observe in Table 1 that by reducing the arrival rate peak by one half reduces the mean queue length peak by one half when E[*S*] = 1. However, we observe in Tables 2 and 3 that as we increase E[*S*], we see a smaller reduction in the peak mean queue length. Certainly we expect the infectious period to be longer than one day for COVID-19 so our theoretical results imply that we need to do more flattening of the curve when patients spend more time infected.

**Table 2:** Comparison between regular and flattened curves peak values and times ($\lambda_{\phi }^{*}=100$).

Curve Type	*τ*	*σ*	E[*S*]	Peak Time	Peak Value
Arrival Curve	10	2	2	10	19.95
Flattened Arrival Curve	20	4	2	20	9.97
Mean Queue Length	10	2	2	11.40	31.29
Flattened Mean Queue Length	20	4	2	21.71	18.20

**Table 3:** Comparison between regular and flattened curves peak values and times ($\lambda_{\phi }^{*}=100$).

Curve Type	*τ*	*σ*	E[*S*]	Peak Time	Peak Value
Arrival Curve	10	2	10	10	19.95
Flattened Arrival Curve	20	4	10	20	9.97
Mean Queue Length	10	2	10	12.93	68.28
Flattened Mean Queue Length	20	4	10	24.51	52.90

**Remark**. *Poisson thinning, when the probabilities for thinning are not a function of time, does not affect the time of the peak arrival rate. The time of the peak load is unaffected whenever the thinning mechanism is also independent of the random service time. In general, we can thin the number of infected individuals appropriately to consider the number of people who enter a hospital to make use of an ICU or not and in either case recover or not*.

*A constant arrival rate over the entire real line generates an M*/*G*/∞ *queue in steady state. The superposition property of Poisson processes and infinite server queues adds a constant to the arrival rate, which does not change these times either. Hence scaling the total number of infected people down, due to flattening the curve, has no effect on the location of the peak mean queue length*.

### 2.3 The *M*_*γ*_(*t*)/*G*/∞ queue with a scaled gamma density arrival rate

We now describe *M*_*γ*_(*t*)/*G*/∞ queueing dynamics when the arrival rate λ_*γ*_(*t*) is a scaled gamma distribution function of time *t* or

$$\begin{array}{c} \lambda_{\gamma }\left(t\right)\equiv \frac{\lambda_{\gamma }^{*}\cdot \beta^{\alpha }}{\Gamma (\alpha )}\cdot t^{\alpha -1}\cdot e^{-\beta t},\;\text{for}\;\text{all}\;t\geq 0\text{,}\;\text{where}\;\Gamma \left(\alpha \right)\equiv \int_{0}^{\infty }e^{-t}t^{\alpha -1}\;dt. \end{array}$$

Here *α* is a shape parameter for the gamma distribution, *β* is the rate parameter for the gamma distribution, and $\lambda_{\gamma }^{*}$ is again the total mean number of infected individuals over the lifetime of the epidemic. Unlike the standard Gaussian case, the gamma density is *only* guaranteed to be bounded when *α* ≥ 1. Since it is unrealistic to assume infinite infection rates, we assume this constraint on *α* for the remainder of this paper. This assumption, implies

$$\begin{array}{c} \underset{-\infty <t<\infty }{\text{max}}\lambda_{\gamma }(t)\leq \lambda_{\gamma }(\frac{\alpha -1}{\beta })=\frac{\lambda_{\gamma }^{*}\cdot \beta }{\Gamma (\alpha )}\cdot {\left(\frac{\alpha -1}{e}\right)}^{\alpha -1}\leq \frac{\lambda_{\gamma }^{*}\cdot \beta }{\sqrt{2\pi \alpha }}, \end{array}$$ (10)

where the final inequality follows from Stirling’s approximation. The properties of gamma densities give us the following integral relationships:

$$\begin{array}{c} \lambda_{\gamma }^{*}=\int_{0}^{\infty }\lambda \left(t\right)\;dt,\;\;\frac{\alpha }{\beta }=\frac{1}{\lambda_{\gamma }^{*}}\int_{0}^{\infty }t\cdot \lambda_{\gamma }\left(t\right)\;dt,\;\;\text{and}\;\;\frac{\alpha }{\beta^{2}}=\frac{1}{\lambda_{\gamma }^{*}}\int_{0}^{\infty }{\left(t-\frac{\alpha }{\beta }\right)}^{2}\cdot \lambda_{\gamma }\left(t\right)\;dt. \end{array}$$

The standard deviation for the spread of the arrival rate distribution is now $\sqrt{\alpha }/\beta$. Hence, we have an upper bound for the peak arrival rate that is inversely proportional to the spread of the arrival rate function. Note that this bound has the same form as for the scaled Gaussian arrival rate case.

One reason for using the gamma density is that it is not symmetric about its mean like the Gaussian one. We have data examples where the arrival rate function is asymmetric and scales to a distribution with a heavy tail. One reason for this is that the number of cases may go towards zero quite slowly i.e. until a vaccine is available in a particular country. These data-driven insights encourage us to explore the scaled gamma density arrival rate function.

**Fig 7:**
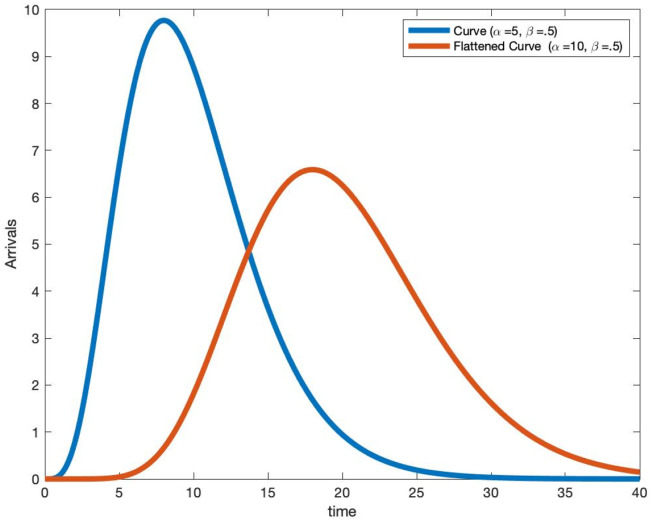
Plot of scaled Gamma density arrival rate curves.

In Fig 7, we plot two scaled gamma density arrival rate functions. The blue curve uses a Gamma(*α* = 5, *β* = .5) density and the red curve (flattened curve) uses a Gamma(*α* = 10, *β* = .5) density. By increasing the variance by a factor of 2, we have reduced the peak value of the arrival rate by a factor of two. We now leverage this analysis of the scaled gamma density arrival rate function, for a Poisson patient arrival model, to obtain new insights from non-stationary queues on the dynamics of the total number infected and their total infection time.

**Theorem 2.11**. *The M*_*γ*_(*t*)/*G*/∞ *queueing model has a Poisson distribution with mean q*(*t*). *Moreover, this mean solves the ordinary differential equation*

$$\begin{array}{cc} \overset{•}{q}\left(t\right)= & \;\frac{\lambda_{\gamma }^{*}\cdot \beta^{\alpha }}{\Gamma (\alpha )}\cdot E[(\alpha -1-\beta \cdot (t-S_{e}))\cdot {\left(t-S_{e}\right)}^{\alpha -2}\cdot e^{-\beta \cdot (t-S_{e})};S_{e}\leq t]\cdot E\left[S\right]\\ = & \;\frac{E[(\alpha -1-\beta \cdot (t-S_{e}))\cdot {\left(t-S_{e}\right)}^{\alpha -2}\cdot e^{-\beta \cdot (t-S_{e})};S_{e}\leq t]}{E[{\left(t-S_{e}\right)}^{\alpha -1}\cdot e^{-\beta \cdot (t-S_{e})};S_{e}\leq t]}\cdot q\left(t\right) \end{array}$$ (11)

*and the solution is given by*

$$\begin{array}{c} q\left(t\right)=\frac{\lambda_{\gamma }^{*}\cdot \beta^{\alpha }}{\Gamma (\alpha )}\cdot E[{\left(t-S_{e}\right)}^{\alpha -1}e^{-\beta \cdot (t-S_{e})};S_{e}\leq t]\cdot E\left[S\right]. \end{array}$$ (12)

*Moreover, for all α* ≥ 1,

$$\begin{array}{c} \underset{-\infty <t<\infty }{\text{max}}q\left(t\right)\leq \frac{\lambda_{\gamma }^{*}\cdot \beta }{\Gamma (\alpha )}\cdot {\left(\frac{\alpha -1}{e}\right)}^{\alpha -1}\cdot E\left[S\right]\leq \frac{\lambda_{\gamma }^{*}\cdot \beta }{\sqrt{2\pi \alpha }}\cdot E\left[S\right]. \end{array}$$

*Proof*. We actually start with the solution. The solution is easily given by the formula from [9]. To find the differential equation, take the derivative of the solution with respect to the time parameter *t*. The resulting bound for the gamma density function yields the corresponding bounds for the mean queue length.

**Theorem 2.12**. *The time of the peak mean queue length solves the following fixed point equation*

$$\begin{array}{c} t^{*}=\frac{\alpha -1}{\beta }+\frac{E[S_{e}\cdot {\left(t^{*}-S_{e}\right)}^{\alpha -2}\cdot e^{-\beta \cdot (t^{*}-S_{e})};S_{e}\leq t^{*}]}{E[{\left(t^{*}-S_{e}\right)}^{\alpha -2}\cdot e^{-\beta \cdot (t^{*}-S_{e})};S_{e}\leq t^{*}]}. \end{array}$$

*Proof*. To find the time of the peak mean queue length, set the time derivative of the mean queue length to zero, i.e.

$$\begin{array}{c} \overset{•}{q}\left(t^{*}\right)=0. \end{array}$$

Using the differential equation given in Eq 11, we then have

$$\begin{array}{c} \frac{\lambda_{\gamma }^{*}\cdot \beta^{\alpha }}{\Gamma (\alpha )}\cdot E[(\alpha -1-\beta \cdot (t^{*}-S_{e}))\cdot {\left(t^{*}-S_{e}\right)}^{\alpha -2}\cdot e^{-\beta \cdot (t^{*}-S_{e})};S_{e}\leq t^{*}]\cdot E\left[S\right]=0. \end{array}$$

Isolating t* by itself on the left hand side gives us the formula.

Note how this result decomposes into a sum of two distinct parts. The first summand is the mode for the arrival rate function, which for the gamma distribution occurs at (*α* − 1)/*β*. The second summand is the positive shift from this mode. This also implies that the time of peak mean queue length lags *after* the time of the peak arrival rate just as in the Gaussian case.

**Fig 8:**
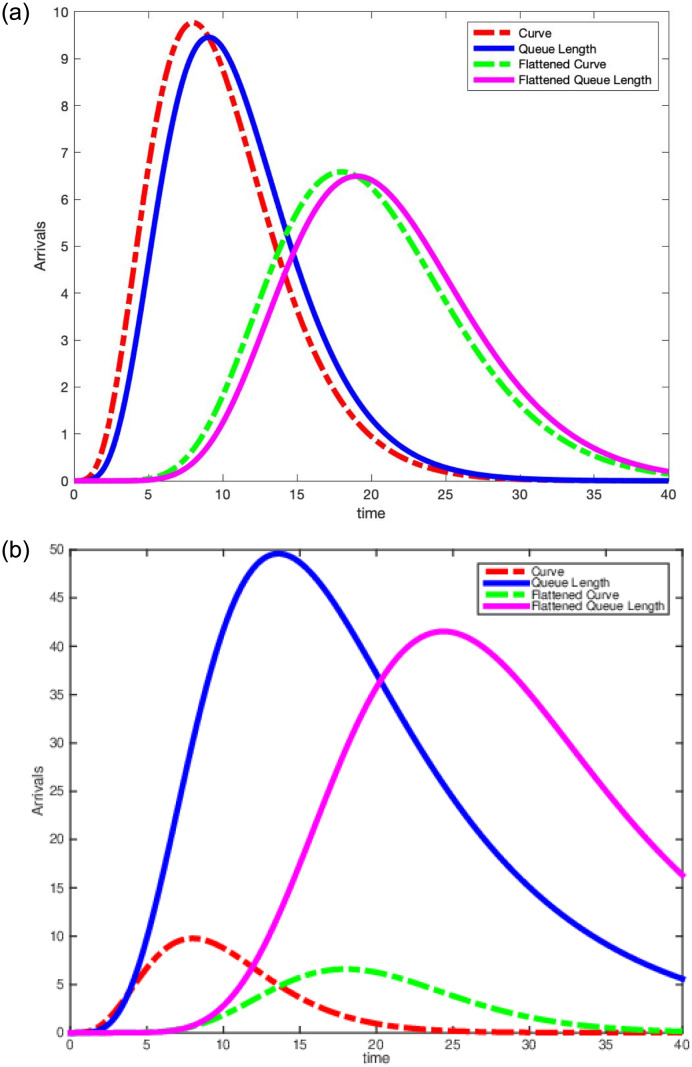
Scaled Gamma density arrival curves and their mean queue lengths E[*S*] = 2 (Left) and E[*S*] = 10 (Right).

Fig 8 plots our scaled gamma density arrival curves with their respective mean queue lengths. On the left of Fig 8, we set the mean service time to be E[*S*] = 1 and on the right of Fig 8, we set the mean service time to be E[*S*] = 10. Finally, we observe in Table 1 that by reducing the arrival rate peak by one half reduces the mean queue length peak by one half when E[*S*] = 1. However, we observe in Tables 4 and 5 that as we increase E[*S*], there is a smaller reduction in the peak mean queue length. We expect the infectious period to be longer than one day for COVID-19, so our theoretical results imply that we may need to do more flattening of the curve as patients spend more time infected.

**Table 4:** Comparison between regular and flattened curves peak values and times.

Curve Type	*α*	*β*	E[*S*]	Peak Time	Peak Value
Scaled Gamma Density Arrival Curve	5	2	1	8.00	9.77
Flattened Scaled Gamma Density Arrival Curve	10	2	1	18.00	6.59
Mean Queue Length	5	2	1	9.06	9.46
Flattened Mean Queue Length	10	2	1	19.03	6.50

**Table 5:** Comparison between regular and flattened curves peak values and times.

Curve Type	*α*	*β*	E[*S*]	Peak Time	Peak Value
Scaled Gamma Density Arrival Curve	5	2	10	8.00	9.77
Flattened Scaled Gamma Density Arrival Curve	10	2	10	18.00	6.59
Mean Queue Length	5	2	10	13.60	49.59
Flattened Mean Queue Length	10	2	10	24.39	41.54

## 3 Understanding COVID-19 through data

**Fig 9:**
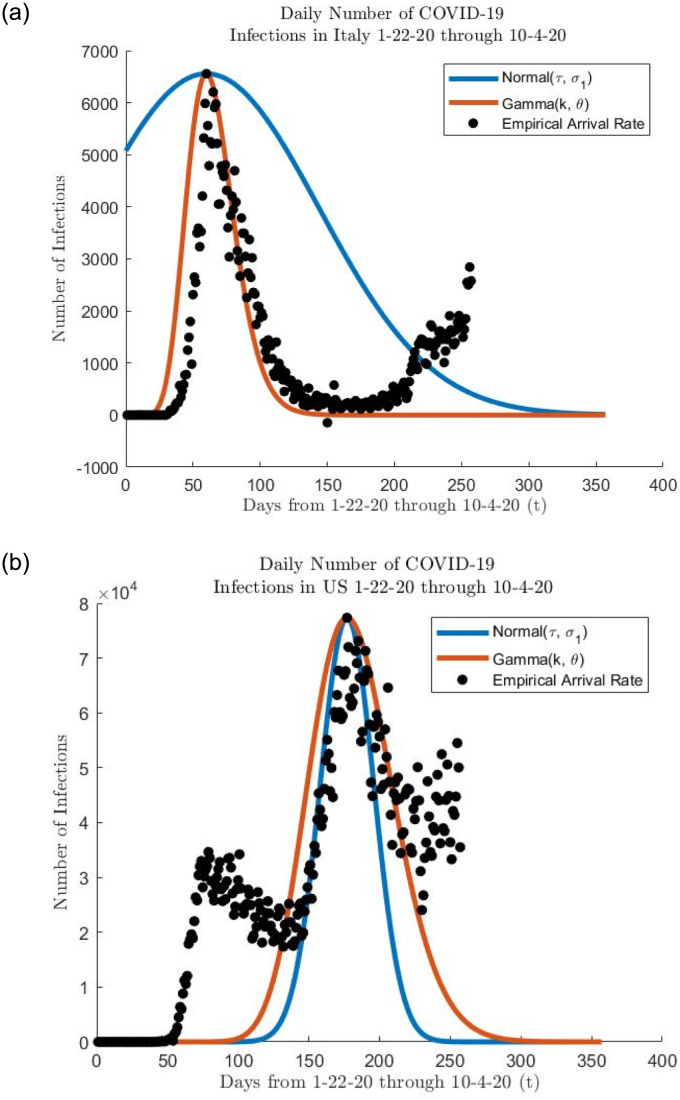
Arrival scatter plots.

In this section, we use some of the infection and death COVID-19 data made available through the Johns Hopkins University website [16]. Fig 9 plots the number of daily confirmed infections in Italy the and United States. Note how the confirmed cases start earlier in Italy than in the United States. Also observe that a local infection rate maximum occurs on the date of April 16th, 2020. For each of the plots, we also overlay properly scaled Gaussian and gamma distribution density functions to get a sense what distribution parameters best fit the data. For Italy, the best scaled Gaussian fit is λ* = 3679154 times a Gaussian(*τ* = 177, *σ* = 19) density and the best scaled gamma fit is λ* = 57824445 times a Gamma(*α* = 36.4, *β* = .2) density. For the United States, the best scaled Gaussian fit is λ* = 1377772 times a Gaussian(*τ* = 60, *σ* = 83) density and the best scaled gamma fit is λ* = 286662 times a Gamma(*α* = 13, *β* = .2) density. Finally, note that we used the “fitdist” function in Matlab for fitting the data to the scaled Gaussian and gamma distributions respectively.

**Fig 10:**
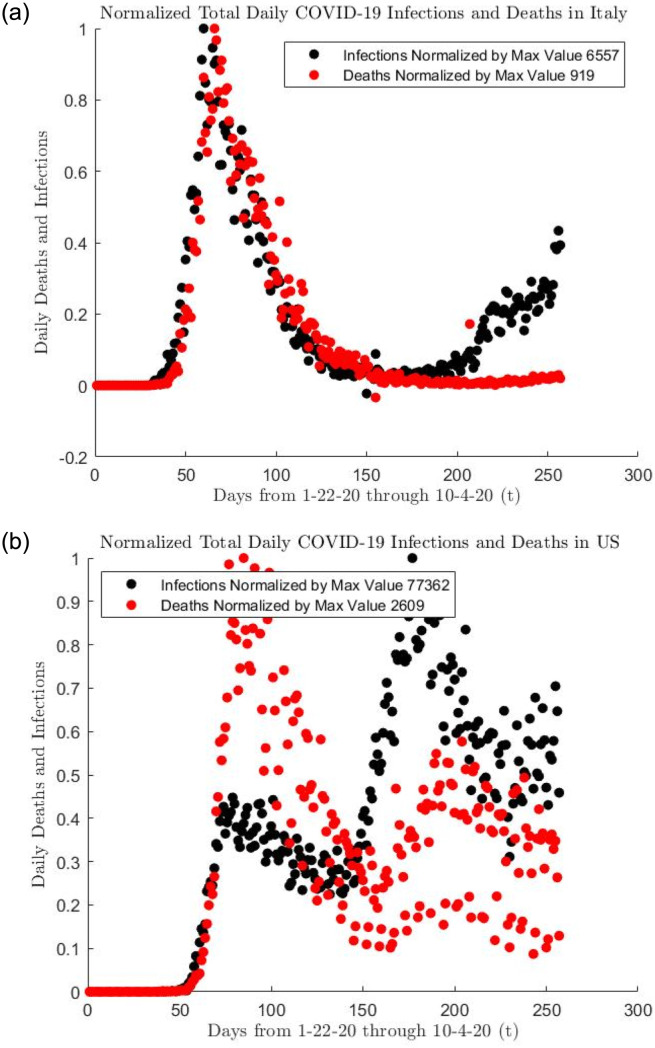
Arrival and death scatter plots.

Fig 10 plots the daily arrivals and deaths from January 22nd, 2020 to October 4th, 2020 normalized by their maximum value (arrival = black dots, deaths = red dots). For Italy and the United States, the maximum confirmed infections was (6557) and (77,362) respectively. Moreover, for Italy, and the United States, the maximum confirmed deaths was (919), and (2609) respectively. Observe that only for the United States does the time for the largest number of deaths lag the same for the largest number of confirmed infections. This is the time lag effect observed in our queueing models. This lag also inspires our construction of a new way to view it.

**Fig 11:**
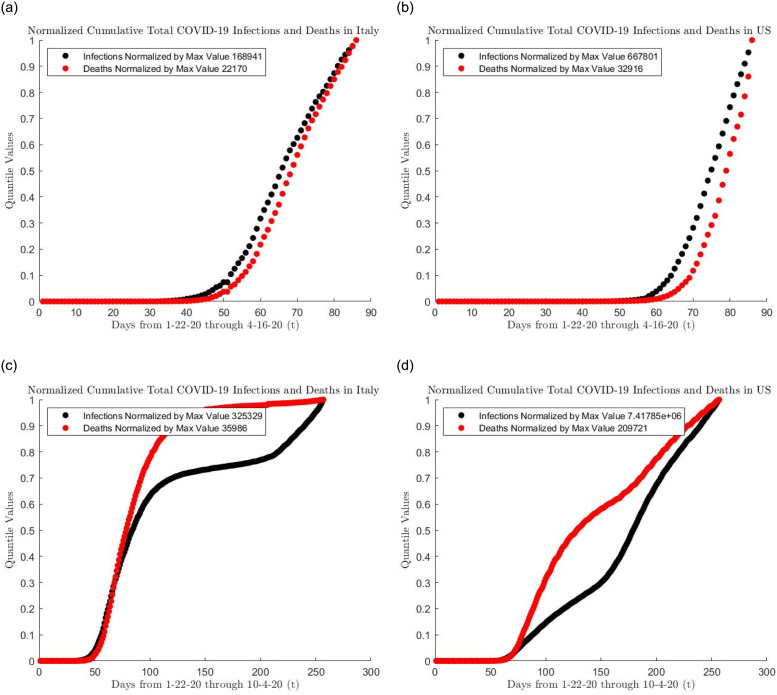
CDF of arrival and death numbers.

Fig 11 plots the cumulative distribution functions for the total number of arrivals and deaths in Italy and the United States. This plot matches the quantiles of the arrivals to the quantiles to that of the deaths. As can be observed from the left-hand plot of Fig 11, the deaths in Italy lagged behind the infections up until April 2020. However the post-April deaths start to slow down and the total death quantiles start leveling off to 1 and the total number of deaths started to lag behind the total number of infections. For the United States, by contrast, the total number of infections overtook the total number of deaths but total death quantiles have not leveled off. Both countries have been able to slow down their death rates but Italy has seemingly done a better job in limiting the number of deaths.

## 4 Key takeaways and insights

The analysis of the queueing model provides both qualitative insights and prescriptive guidance on how to flatten the demand for hospital resources.

### 4.1 Flattening the curve

First, we show that the peak number of hospitalized patients is inversely related to flattening the rate of arriving patients (Theorem 2.1). This insight provides a mechanism to control the peak number of hospitalized people through the standard deviation parameter *σ*. As a result, flattening should be inversely proportional to the amount of hospital capacity to avoid overcrowding. However, the effectiveness of social distancing on flattening the curve, i.e. increasing *σ*, remains an open question. Additionally, we find that flattening the curve has a smaller impact on the peak number of patients when the service times are long. This means the longer patients stay in the hospital, the more society must flatten the curve in order to achieve the same capacity level. In the three examples with an exponential service distribution and a scaled Gaussian density arrival rate, we flattened the peak arrival rate by 50%, however, we see that the peak mean queue length was reduced by 47%, 42%, 23% respectively for E[*S*] equaling 1, 2, and 10. For the scaled gamma density arrival rate setting, a 33% reduction in the peak arrival rate obtains a 31% and 16% reduction respectively for E[*S*] = 1 and E[*S*] = 10.

### 4.2 Time lag between the peak infection rate and the peak number of patients

A key takeaway from our queueing analysis is that there is a lag between the time of the peak arrival rate of infected patients does not coincide with the time of the peak of number of hospitalized people (or deaths). We characterize this time lag exactly for the simplified queue model (Theorem 2.2). First, we show that the peak number of infected lags behind the peak number of newly infected individuals. Surprisingly, there is a nonlinear relationship between flattening the curve and the time lag between the peak rate of newly admitted patients and the peak demand for hospital resources (Theorem 2.2). Moreover, we show in Theorem 6.3 that the time lag *ℓ* is bounded by the mean service time for an exponential distribution i.e.

$$\begin{array}{c} \frac{1}{\mu }-\frac{1}{\mu^{3}\sigma^{2}+\mu }=\frac{1}{\mu }\cdot \frac{{\left(\mu \sigma \right)}^{2}}{{\left(\mu \sigma \right)}^{2}+1}\leq \ell \leq \frac{1}{\mu }=E\left[S\right]. \end{array}$$

This analysis also implies that as we flatten the curve i.e. increase *σ*, the time lag converges to the mean service time 1/*μ*.

## 5 Conclusion

In this paper, we present a simple infinite server queueing model for the number of infected people with Covid-19. This analysis can be used to predict how and when many people will become infected, need a hospital bed, or need a ventilator in the near future. We show how the dynamics of the number infected explicitly depends on the arrival rate function. We explore the case of a symmetric rate function by using a scaled Gaussian density function. Our asymmetric example is a scaled gamma density function. We explicitly calculate for both cases the times of their peak mean queue lengths and the peak mean queue lengths, along with their dependence on the duration of infection.

To address more of the health operations issues for the larger pandemic event, we outline below some of the remaining important questions that still need to be studied. By leveraging operations research tools and techniques, hospitals have prided themselves on the efficiency that comes with just-in-time supply management and minimizing empty beds, thus saving on hospital costs. However as a result, they are often not equipped for an epidemic surge such as this one. Despite our efforts to understand the impact of COVID-19 on the healthcare system, there is plenty of additional research that needs to be done in order to understand these emerging complexities.

One area of importance is to understand the effects of surges in arrivals into the healthcare system. Some recent research has studied queueing models with self-exciting point processes that model random surges in demand, see for example [15, 17–21]. These types of models would generalize our model in this paper but they are more complicated and would disguise the easy, back of the envelope insights from simpler models.

Future research should address the impact of testing and time lags in testing for the coronavirus. It has been shown through empirical analyses that testing delays can have a huge impact on the reported numbers of positive cases and health outcomes, see for example [22]. Our density plots in Fig 11 show that testing is an important indicator for predicting deaths to follow.

Finally, we are observing in some cities that it is important to use demographic information for understanding the impact of COVID-19 on particular communities or regions of the country. In the city of Chicago, despite African-Americans making up roughly 30% of the Chicago population, African-Americans make up 52% of cases of COVID-19 and 68% of COVID-19 deaths, see for example [23]. Also in NYC, it has been reported that COVID-19 has a disproportionately negative impact on low-income communities [24]. Recent work by [25] combines queueing theory with demographic information to get a deeper understanding of blood donation dynamics. This suggests that similar demographic analyses are also needed to understand the full impact of COVID-19 on marginalized populations.

## 6 Appendix

### 6.1 The *M*_φ_(*t*)/*G*/∞ queue

#### 6.1.1 Proof of Theorem 2.2

*Proof*. In order to find the peak mean queue length we need to set the time derivative of the mean queue length to zero i.e.

$$\begin{array}{c} \overset{•}{q}\left(t^{*}\right)=0. \end{array}$$

Now using the differential equation given in Eq 2, we have

$$\begin{array}{c} \overset{•}{q}\left(t^{*}\right)=E[\left(t^{*}-\tau -S_{e})\cdot \varphi (\frac{t^{*}-\tau -S_{e}}{\sigma }\right)]=0. \end{array}$$

This implies that

$$\begin{array}{ccc} \left(t^{*}-\tau )\cdot E[\varphi (\frac{t^{*}-\tau -S_{e}}{\sigma })\right] & = & E[S_{e}\cdot \varphi (\frac{t^{*}-\tau -S_{e}}{\sigma })]. \end{array}$$

Now isolate t* by itself to get

$$\begin{array}{cc} t^{*} & =\tau +E[S_{e}\cdot \varphi (\frac{t^{*}-\tau -S_{e}}{\sigma })]\cdot E{\left[\varphi (\frac{t^{*}-\tau -S_{e}}{\sigma })\right]}^{-1}\\ & =\tau +\frac{\lambda_{\varphi }^{*}\cdot E\left[S\right]}{\sigma \cdot q(t^{*})}\cdot E[S_{e}\cdot \varphi (\frac{t^{*}-\tau -S_{e}}{\sigma })]. \end{array}$$

This completes the proof.

#### 6.1.2 Discrete service distribution

**Corollary 6.1**. *The mean behavior for the M*_*φ*_(*t*)/*D*_*n*_/∞ *queueing model is the solution to the ordinary differential equation*

$$\begin{array}{ccc} \overset{•}{q}\left(t\right) & = & \sum_{i=1}^{n}p_{i}\cdot (\lambda_{\varphi }\left(t\right)-\lambda_{\varphi }\left(t-\Delta_{i}\right))\\ & = & \sum_{i=1}^{n}p_{i}\cdot (\frac{\lambda_{\varphi }^{*}}{\sigma }\cdot \varphi (\frac{t-\tau }{\sigma })-\frac{\lambda_{\varphi }^{*}}{\sigma }\cdot \varphi (\frac{t-\tau -\Delta_{i}}{\sigma })) \end{array}$$ (13)

*and the solution is given by*

$$\begin{array}{ccc} q(t) & = & \sum_{i=1}^{n}p_{i}\cdot (\int_{t-\Delta_{i}}^{t}\frac{\lambda_{\varphi }}{\sigma }\cdot \varphi (\frac{s-\tau }{\sigma })ds)\\ & = & \sum_{i=1}^{n}p_{i}\cdot \lambda_{\varphi }\cdot (\Phi (\frac{t-\tau }{\sigma })-\Phi (\frac{t-\Delta_{i}-\tau }{\sigma })). \end{array}$$ (14)

*Proof*. The proof follows the thinning of Poisson processes and the expression given in Eq 6.

#### 6.1.3 Exponential service distribution

Theorem 2.10

*Proof*. First we observe that from Theorem 2.2 that t* solves the following fixed point equation

$$\begin{array}{ccc} t^{*} & = & \tau +E[S_{e}\cdot \varphi (\frac{t^{*}-\tau -S_{e}}{\sigma })]\cdot E{\left[\varphi (\frac{t^{*}-\tau -S_{e}}{\sigma })\right]}^{-1}. \end{array}$$

If we define the peak lag time to be the parameter *ℓ*, we then have *ℓ* = t* − *τ* and the following fixed point equation

$$\begin{array}{c} \ell =E[S_{e}\cdot \varphi (\frac{\ell -S_{e}}{\sigma })]\cdot E{\left[\varphi (\frac{\ell -S_{e}}{\sigma })\right]}^{-1}. \end{array}$$

Thus, if we define X to be a standard (mean 1) exponential random variable, then we have

$$\begin{array}{cc} \ell & =E[S_{e}\cdot \varphi (\frac{\ell -S_{e}}{\sigma })]\cdot E{\left[\varphi (\frac{\ell -S_{e}}{\sigma })\right]}^{-1}\\ & =E[\frac{X}{\mu }\cdot \varphi (\frac{\ell -X/\mu }{\sigma })]\cdot E{\left[\varphi (\frac{\ell -X/\mu }{\sigma })\right]}^{-1}\\ & =\frac{\mu \sigma \cdot e^{-\mu \ell }\cdot e^{\mu^{2}\ell^{2}/2}\cdot (\left(\ell -\mu \sigma^{2})\cdot \Phi (\frac{\ell }{\sigma }-\mu \sigma )+\sigma \cdot \varphi (\frac{\ell }{\sigma }-\mu \sigma \right))}{\mu \sigma \cdot e^{-\mu \ell }\cdot e^{\mu^{2}\ell^{2}/2}\cdot \Phi (\frac{\ell }{\sigma }-\mu \sigma )}\\ & =\ell -\mu \sigma^{2}+\sigma \cdot \varphi (\frac{\ell }{\sigma }-\mu \sigma )\cdot \Phi {\left(\frac{\ell }{\sigma }-\mu \sigma \right)}^{-1} \end{array}$$

Subtracting the time lag *ℓ* from both sides and using the *ψ* function gives us

$$\begin{array}{c} \mu \sigma =\sigma \cdot \varphi (\frac{\ell }{\sigma }-\mu \sigma )\cdot \Phi {\left(\frac{\ell }{\sigma }-\mu \sigma \right)}^{-1}\;\;\;\text{if}\;\text{and}\;\text{only}\;\text{if}\;\;\;\psi (\mu \sigma )=\frac{\ell }{\sigma }-\mu \sigma . \end{array}$$

This gives us an explicit solution for *ℓ* which is

$$\begin{array}{c} \ell =\sigma \cdot (\mu \sigma +\psi (\mu \sigma )). \end{array}$$

Now it remains to prove two identities from the next lemma.

**Lemma 6.2** *We have the following two identities for standard exponential random variables*:

$$\begin{array}{c} E[\varphi (\frac{\ell -X/\mu }{\sigma })]=\mu \sigma \cdot e^{-\mu \ell }\cdot e^{\mu^{2}\ell^{2}/2}\cdot \Phi (\frac{\ell }{\sigma }-\mu \sigma ) \end{array}$$

*and*

$$\begin{array}{cc} E & \left[\frac{X}{\mu }\cdot \varphi (\frac{\ell -X/\mu }{\sigma })\right]\\ & =\mu \sigma \cdot e^{-\mu \ell }\cdot e^{\mu^{2}\ell^{2}/2}\cdot (\left(\ell -\mu \sigma^{2})\cdot \Phi (\frac{\ell }{\sigma }-\mu \sigma )+\sigma \cdot \varphi (\frac{\ell }{\sigma }-\mu \sigma \right)). \end{array}$$

*Proof*. For the first equality, define G to be a standard (mean 0 and variance 1) Gaussian random variable. Exploiting the version of Stein’s lemma as given in [21, 26] for standard Gaussian random variables G along with its analogue for exponential random variables, we have for all suitable functions *f*

$$\begin{array}{c} E[G\cdot f(G)]=E[f^{\prime }\left(G\right)]\;\;\;\text{and}\;\;\;E[f(X)]=f\left(0\right)+E[f^{\prime }\left(X\right)]. \end{array}$$

Assuming that G and X are independent random variables and applying Stein’s formula to generalized functions, gives us

$$\begin{array}{ccc} E[\varphi (\frac{\ell -X/\mu }{\sigma })] & = & E[E[G\cdot \{G>\frac{\ell -X/\mu }{\sigma }\}|X]]\\ & = & E[E[G\cdot \{G>\frac{\ell -X/\mu }{\sigma }\}|G]]\\ & = & E[G\cdot P\{G>\frac{\ell -X/\mu }{\sigma }|G\}]. \end{array}$$

Since X is a standard exponential, we use Stein’s formula again and obtain

E[φ(ℓ-X/Xμμσ)]=E[G·P{X>μ·(ℓ-σG)|G}]=E[G·(e-μ·(ℓ-σG)·{G≤ℓσ}+{G≤ℓσ}¯)]=E[G·(e-μ·(ℓ-σG)-1)·{G≤ℓσ}]=E[μσ·e-μ·(ℓ-σG)·{G≤ℓσ}].

Finally, Girsanov’s formula gives us

E[φ(ℓ-X/Xμμσ)]=μσ·e-μℓ·E[eμσG]·P{G+μσ≤ℓσ}=μσ·e-μℓ·eμ2σ2/2·Φ(ℓσ-μσ).

Since

$$\begin{array}{cc} \frac{d\;}{d\ell }E[\varphi (\frac{\ell -X/\mu }{\sigma })]= & \frac{1}{\sigma }\cdot E[\varphi^{\prime }(\frac{\ell -X/\mu }{\sigma })]\\ = & \frac{1}{\sigma }\cdot (-\frac{\ell }{\sigma }\cdot E[\varphi (\frac{\ell -X/\mu }{\sigma })]+\frac{1}{\sigma }\cdot E[\frac{X}{\mu }\cdot \varphi (\frac{\ell -X/\mu }{\sigma })]), \end{array}$$

we now have

$$\begin{array}{cc} \frac{d\;}{d\ell }\text{log}E[\varphi (\frac{\ell -X/\mu }{\sigma })] & =\frac{d\;}{d\ell }E[\varphi (\frac{\ell -X/\mu }{\sigma })]\cdot E{\left[\varphi (\frac{\ell -X/\mu }{\sigma })\right]}^{-1}\\ & =-\frac{\ell }{\sigma^{2}}+\frac{1}{\sigma^{2}}\cdot E[\frac{X}{\mu }\cdot \varphi (\frac{\ell -X/\mu }{\sigma })]\cdot E{\left[\varphi (\frac{\ell -X/\mu }{\sigma })\right]}^{-1}. \end{array}$$

Taking the logarithmic derivative for the right hand side of the first identity and then multiplying both sides by *σ*^2^ gives us

$$\begin{array}{cc} -\mu \sigma^{2}+\sigma \cdot \varphi (\frac{\ell }{\sigma }-\mu \sigma ) & \cdot \Phi {\left(\frac{\ell }{\sigma }-\mu \sigma \right)}^{-1}\\ & =-\ell +E[\frac{X}{\mu }\cdot \varphi (\frac{\ell -X/\mu }{\sigma })]\cdot E{\left[\varphi (\frac{\ell -X/\mu }{\sigma })\right]}^{-1}. \end{array}$$

We then have

$$\begin{array}{cc} E & \left[\frac{X}{\mu }\cdot \varphi (\frac{\ell -X/\mu }{\sigma })\right]\\ & =E[\varphi (\frac{\ell -X/\mu }{\sigma })]\cdot (\ell -\mu \sigma^{2}+\sigma \cdot \varphi (\frac{\ell }{\sigma }-\mu \sigma )\cdot \Phi {\left(\frac{\ell }{\sigma }-\mu \sigma \right)}^{-1}) \end{array}$$

and the second identity follows from using the first.

**Theorem 6.3** *For the function ψ*(*x*), *we have the following bound*.

$$\begin{array}{c} -\frac{1}{x^{3}+x}\leq \psi \left(x\right)+x-\frac{1}{x}\leq 0. \end{array}$$

*Thus, we have in the exponential service setting with a scaled Gaussian density arrival rate function that the time lag is bounded by*

$$\begin{array}{c} -\frac{1}{\mu }\cdot \frac{1}{{\left(\mu \sigma \right)}^{2}+1}\leq \ell -\frac{1}{\mu }\leq 0. \end{array}$$

*Proof*. The first inequality follows from Theorem 7.2 in [14]. Therefore, the time lag bounds follow by substituting *x* = *σμ*.

#### 6.1.4 Hyper-exponential service distribution

Another continuous distribution of interest is the hyper-exponential distribution, a special case of a phase type distribution. When holding the mean of the distribution fixed, the Erlang distribution always has a smaller variance than the corresponding one for an exponential distribution. The corresponding hyper-exponential distribution always has a larger variance than both of theirs. Usually the hyper-exponential distribution is determined by two *n*-dimensional vectors of parameters (*p*_1_, *p*_2_, …, *p*_*n*_) and (*μ*_1_, *μ*_2_, …, *μ*_*n*_). The first vector represents the probabilistic weights for an exponential distribution associated with the corresponding rate parameter given by the second vector. The hyper-exponential density is then a convex combination of exponential distributions.

**Corollary 6.4**. *The M*_*φ*_(*t*)/*H*_*n*_/∞ *queue with a scaled Gaussian arrival rate is given by the following closed form expression for the mean transient queue length*

$$\begin{array}{c} q_{H_{n}}\left(t\right)=\sum_{i=1}^{n}p_{i}\cdot q_{i}\left(t\right), \end{array}$$

*where*

$$\begin{array}{c} q_{i}\left(t\right)=\sigma \cdot e^{-\mu_{i}\cdot \left(t-\tau \right)}\cdot e^{\mu_{i}^{2}\sigma^{2}/2}\cdot \Phi (\frac{t-\tau }{\sigma }-\mu_{i}\cdot \sigma ) \end{array}$$

*for all i* = 1, …, *n*.

*Proof*. This follows from the fact that the hyper-exponential is just a thinning of a Poisson process. Thus, the sum is also a Poisson process with the sum of the rates.

**Corollary 6.5**. *The M*_*φ*_(*t*)/*H*_*n*_/∞ *queue with arrival rate given by a Gaussian distribution with parameters has the following closed form expression for the time lag*

$$\begin{array}{c} \ell =\frac{\sum_{j=1}^{n}\alpha_{j}\mu_{j}\cdot e^{-\mu_{j}\ell }\cdot e^{\mu_{j}^{2}\cdot \sigma^{2}/2}\cdot \Phi (\frac{\ell }{\sigma }-\mu_{j}\cdot \sigma )}{\sum_{j=1}^{n}\alpha_{j}\mu_{j}\cdot e^{-\mu_{j}\cdot \ell }\cdot e^{\mu_{j}^{2}\cdot \sigma^{2}/2}\cdot (\left(\ell -\mu_{j}\cdot \sigma^{2})\cdot \Phi (\frac{\ell }{\sigma }-\mu_{j}\cdot \sigma )+\sigma \cdot \varphi (\frac{\ell }{\sigma }-\mu_{j}\cdot \sigma \right))} \end{array}$$

*where*

$$\begin{array}{c} \alpha_{j}\equiv \frac{p_{j}}{\mu_{j}\cdot \sum_{i=1}^{n}p_{i}/\mu_{i}}. \end{array}$$

*Proof*. This follows from the fact that the hyper-exponential is just a thinning of a Poisson process that is the superposition of the sum of the rates. Thus, the sum is also a Poisson process with the sum of the rates. Moreover, the stationary excess distribution of a hyper-exponential is another hyper-exponential distribution with modified rates [11].
